# High-Tech and Nature-Made Nanocomposites and Their Applications in the Field of Sensors and Biosensors for Gas Detection

**DOI:** 10.3390/bios10110176

**Published:** 2020-11-13

**Authors:** Daniele Zappi, Matiss Martins Ramma, Viviana Scognamiglio, Amina Antonacci, Gabriele Varani, Maria Teresa Giardi

**Affiliations:** 1 Istituto di Cristallografia, CNR Area Della Ricerca di Roma, 00015 Monterotondo Scalo Rome, Italy; daniele.zappi@ic.cnr.it (D.Z.); viviana.scognamiglio@ic.cnr.it (V.S.); amina.antonacci@ic.cnr.it (A.A.); 2 Biosensor Srl, Via Degli Olmetti 44, 00060 Formello Rome, Italy; m.ramma@biosensor.it (M.M.R.); g.varani@biosensor.it (G.V.)

**Keywords:** gas sensors, biosensors, nanomaterials, metal oxides, conductive polymers, enzymes, enzymatic inhibition, multi-enzyme cycle, whole cells biosensors, bio-sniffers

## Abstract

Gas sensors have been object of increasing attention by the scientific community in recent years. For the development of the sensing element, two major trends seem to have appeared. On one hand, the possibility of creating complex structures at the nanoscale level has given rise to ever more sensitive sensors based on metal oxides and metal–polymer combinations. On the other hand, gas biosensors have started to be developed, thanks to their intrinsic ability to be selective for the target analyte. In this review, we analyze the recent progress in both areas and underline their strength, current problems, and future perspectives.

## 1. Introduction

Gas sensing technology is undergoing continuous research and development in recent years [1]. The need to monitor ever-smaller amounts of volatiles in work ambient air and the environment has pushed both for the miniaturization and the betterment of the limit of detections of such sensors.

Concerning the nature of the sensing element, two major trends have appeared: first, the possibility to create complex structures at the nanoscale level has given rise to ever more sensitive sensors, based on metal oxides and metal–polymer combinations such as zinc oxide and polyaniline hybrid materials that have often synergetic properties from both elements when combined in a single material. Secondly, gas biosensors have started to be developed, thanks to the intrinsic ability of the biological recognition element to be more selective toward the target analyte.

Gas sensors are typically categorized as chemical sensors, and they are composed of a sensing element that reacts when exposed to one or more target gasses, creating a physicochemical variation, which is associated with a transducer that converts the above-mentioned physicochemical variation into an electrical, measurable signal. In gas sensors, the two most investigated aspects are sensitivity and selectivity toward target gas [2]. Both are core points for the evaluation of the quality of the developed gas sensors.

One point of note is that while gas sensor technology has become increasingly advanced when using inorganic materials as sensing layers, sparse research has been done up to now in the development of gas sensors having a bio-based sensing layer (e.g., enzymes, nucleic acids, or living organisms). However, it is expected that research efforts aimed at biological gas sensing systems will increase in the future, especially in the framework of improved sensitivity and selectivity.

Selectivity, in particular, is a severe limiting factor for gas sensor applications as it limits the optimal working conditions of the sensor. Furthermore, sensitivity toward target gas is also a challenge. These problems have been addressed in many ways in recent non-biological gas sensors by incorporating more complex solutions as sensing elements. Hybrid gas sensing platforms such as thin films, special dopants, and a wide range of nanomaterials have all found use in the development of innovative sensing solutions. On the other hand, the results of biological sensors have also show shown great promises as their tunability for target gases and the detection of low gas concentration have been remarkable.

Considering the availability of comprehensive reviews on inorganic-based gas sensors present in the literature [3,4,5,6,7], the present review aims to investigate the most recent advances regarding bio-based gas sensors and compare them with their inorganic counterparts to provide a clear outlook on the state of the art of the research for future perspectives.

In the next section, we will provide an overview of some recent advances in the field of gas sensors based on inorganic nanomaterial, both alone and in conjunction with conductive polymers. Then, common production strategies for nanomaterials, their pros and cons, and what nanomaterial type can each produce will be discussed. Then, the review will move to gas biosensors, recent progress, and common strategies to entrap the sensing element near the transductor. Finally, a comparison of the two typologies of sensor will be performed, underlining the strengths and weakness of each class.

## 2. Gas Sensors Based on Nanomaterials

Non-biological material-based gas sensors employ a wide class of materials. Nanostructured materials such as nanowires and nanorods have been intensively studied owing to their potential technological applications in gas sensing. Semiconductors such as ZnO and SnO_2_ have shown excellent properties for gas monitoring when used in nanomaterial form. In particular, ZnO nanowires provide a direct bandgap (3.37 eV) and a large excitation binding energy (60 meV), which makes them suitable for utilization in gas sensor devices [8]. On the other hand, tetragonal rutile nanocrystalline SnO has been used to develop a room-temperature gas sensor for H_2_ [9].

The functioning of gas sensors based on nanostructured materials can be largely explained when considering electron distribution in the surface layer of the nanostructure: in clean air condition, there is an abundance of oxygen, which tends to attract the electrons of the surface layer, preventing electrical conductibility. When the nanostructured material is exposed to the target gas, which is often a reducing one, electrons are free to move in the surface layer of the nanomaterial, resulting in reduced electrical resistance.

In the following, the sensors are classified based on the transduction mechanism.

- *Conductivity-based sensors*

In this type of sensor, a change in conductivity of the sensing layer following exposure to the target gas is measured. The sensitivity of the sensor toward the target gas has been expressed using the following equation:

$$Sensitivity\;\Delta R\left(\%\right)=\frac{R_{g}-R_{a}}{R_{a}}\times \;100$$

where *R_g_* and *R_a_* are sensors resistance in gas and air.

Benzaldehyde gas sensors were fabricated using NiO nanomaterials, which were synthesized from two different precursors (nitrate and chloride precursor solutions) and grown in air and N_2_ growth environments [10]. The resulting nanostructures showed variations in shape and gas-sensing abilities, but all changes in resistivity upon detection peaked around 300 °C for all nanostructures with benzaldehyde at a fixed concentration of 100 ppm. The sensitivity reached 182.2% (air) and 209.4% (N_2_) for nitrate solution precursor synthesized nanostructures and 242.0% (air) and 249.5% (N_2_) for nanostructures grown with a chloride solution precursor. A larger increase in temperature resulted in a sharp drop of sensitivity, so further investigation of these sensors was followed up at 300 °C. Selectivity was tested on various gases at fixed 100 ppm concentrations, such as ethanol, acetone, benzene, toluene, benzyl alcohol, methanol, and ammonium hydroxide. In comparison to benzaldehyde, the results showed a sensitivity for benzene that was about four times smaller, and for methanol, it was two times smaller, highlighting that these devices are highly selective toward benzaldehyde. Reproducibility and stability were also tested by eight cycles of injecting and removing 15 ppm benzaldehyde gas, and the results showed that the resistance peak values were virtually constant. Further shelf life studies with repeated measurements of benzaldehyde 100 ppm every 10 days showed that only a small 1.44–2.44% resistance decrease was noticed after 40 days, indicating that these sensors are highly stable.

Gas sensors for air and O_3_ detection based on changes in the system conductivity were assembled using Mg-doped and undoped In_2_O_3_ film [11]. The research showed that by decreasing bulk conductivity contribution to the overall one makes the conductivity of the surface the biggest contributor. This resulted in a more responsive sensor when exposed to gas analytes. Measurements carried out comparing Mg-doped and undoped In_3_O_2_ textured films response in the O_3_ gas range of 0.05–0.150 ppm showed that the sensor response for the doped thin film samples was around two times larger when compared to the undoped ones. However, overly doped samples would also start experiencing decreasing sensitivity to these gases due to the depletion of the surface electron accumulative layer (SEAL) of the In_3_O_3_ films, which is the contributing factor for the surface conduction. The sensors were able to monitor O_3_ concentrations at an extremely low concentration of 50 ppb.

Volatile compounds such as dimethyl disulfide (DMDS) and 1-butanol are early indicators for microbial infections for dry ham and potatoes, and the detection of these compounds can be used for the quality control of food products [12]. The authors demonstrated a working dimethyl disulfide and 1-butanol sensor based on different kinds of zinc oxide and tin dioxide, which were fabricated by applying ZnO/TiO_2_ on gold interdigitated gold electrodes to detect the target analytes by measuring changes in the electrical conductivity of the sample. Different types of ZnO and TiO_2_ materials were tested, such as TiO_2_ thin films with thicknesses of 150 and 940 nm, individual ZnO and TiO_2_ thick films, as well as composite materials of ZnO/TiO_2_ with ZnO/TiO_2_ ratios of 75/25, 50/50, and 25/75%. Experiments for the detection of DMDS were carried out in the range of 1–100 ppm, and the results showed that composite materials with a ratio of 50/50% were superior with 1265 and 5583% change in conductivity for 1 and 10 ppm of DMDS, respectively. TiO_2_ thick films showed a conductivity change of 550, 3639% whilst ZnO thick films had a conductivity change of 643 and 3930%. Meanwhile, 150 nm thin film showed much smaller changes of 10,39% whilst 950 nm film had slightly better results of 95 and 457%.

The authors compared the obtained data with those obtained using the most sensitive commercially available sensor, “Figaro TGS 822” (U.S. Pat No. 9,182,366 B2), which showed changes of 47 and 180% when exposed to similar gas concentrations. The researchers pointed out that extensive exposure to DMDS at 100 ppm had detrimental sensitivity loss, so experiments were not carried out extensively at this concentration. Individual ZnO and TiO_2_ thick film component sensors reached 95% of the maximum in 2 min and had a recovery time of 3 min. Commercially available Figaro sensors had similar behavior as thick films, but TiO_2_ thin films had a faster response and recovery time. The reproducibility of DMDS gas sensors was tested by refabricating the sensors and retesting them at the same DMDS concentrations, and results showed that the lowest reproducibility was for the thin films for which the response differed by up to 30%, ZnO/TiO_2_ composites differed by up to 10–20%, and the differences in response for commercial sensors was found to be up to 20%. The rest of the sensors were found to be more reproducible with values differing only about ±10%. It is worth mentioning that the fabricated sensors had a substantially lower detection limit for DMDS with values ranging from 0.01 to 0.05 ppm, but although the sensor response and response profiles for identical sensors were found to be quite similar, the baseline currents or resistivities of the sensors were found to differ quite substantially when refabricated. For example, the initial resistance values for thin films varied up to 100% with 150 nm thin films having the most substantial resistance variation and 25–40% for the other types of sensors. The reproducibility issues for thin films is believed to be affected by large changes to the relative surface roughness in the fabrication process when compared to the thin film thicknesses themselves. Long-term stability tests were also carried out, showing that the baseline resistivity varied around 10% every day. All the before-mentioned gas sensing materials were also tested for 1-butanol vapor detection at concentrations ranging from 1 to 100 ppm, and they were found to be less accurate when compared to sensing the DMDS with lower limit detection concentrations being 0.025–0.100 ppm. For ppm values of 1, 10, and 100 ppm, the highest percentage changes to conductivity were again found to be for composite materials of ZnO/TiO_2_, with a 50/50% mix ratio with values being 245, 1630, and 2835%, respectively. ZnO thick film sensors were substantially more sensitive than TiO_2_, with conductivity changes being 174, 1224, and 1624% for ZnO and 155, 615, and 783% TiO_2_ thick films. Thin films were much less sensitive, as the changes were 6, 25, and 42% for 150 nm and 31, 203, and 390% for 940 nm thin films. The highest sensing commercial sensor in this study was again TGS 822 that had changes of conductivity of 100, 221, and 743%. Interestingly enough, contrary to DMDS sensors, butanol sensors reached their saturation response at above 100 ppm, but the reproducibility, baseline resistivity, response, and recovery dynamics were very similar to DMDS.

- *Optical-based sensors*

**Figure 1:**
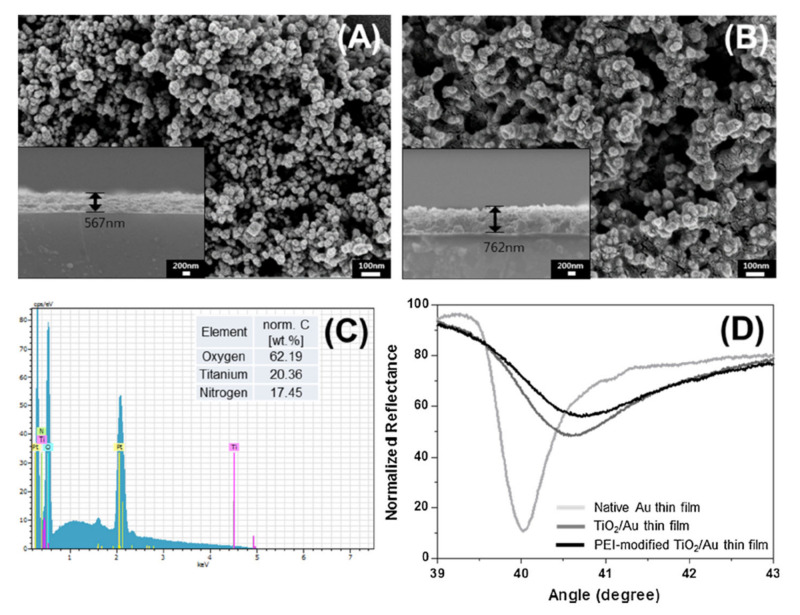
SEM images of the modified thin films developed ((**A**) and (**B**)), elemental analysis (**C**), and result obtained with the surface plasmon resonance (SPR) technique for the detection of formaldehyde (**D**). Reproduced from [13] with permission from Elsevier (License number 4927020376869).

TiO_2_/Au polymer modified thin films were used to create an advanced type of formaldehyde gas sensor [13]. This particular gas sensing material was used to detect low amounts of formaldehyde interacting on the surface of the material using the optical-based detection method of surface plasmon resonance (SPR). This technique involves the use of a light source aimed at a specific resonance angle to the surface of the sample to excite the surface plasmons, in this case, the target material being the polymer-modified TiO_2_/Au thin films. Figure 1A shows the surface images of TiO_2_ nanoparticles on the Au films before polymer modification, while Figure 1B shows nanoparticles after polymer modification and the subsequent increase in film thickness from 567 to 762 nm. In Figure 1C, the elemental analysis of NH_3_ sensing TiO_2_/Au polymer-modified thin film is reported, while Figure 1D depicts the comparison among native Au, TiO_2_/Au, and TiO_2_/Au polymer-modified thin films concerning their relative reflectance of light at various SPR resonance angles.

Then, these intensity measurements of the reflective light can be exploited for the detection of formaldehyde with a detection limit of 0.2 ppm and a linear range from 0.2 to 1.8 ppm and the upward detection limit being around 3.5 ppm. In particular, the variation of light reflected at a specific angle was correlated with the concentration of formaldehyde following the equation:

y=5.978x+1.233

where *x* is the concentration of the formaldehyde gas, expressed in ppm, while *y* is the change in reflectance at fixed angle, expressed in relative units.

The study points out that this detection range can have its applications for the detection of biomarkers in patient breath, such as formaldehyde, which can be used to diagnose breast cancer, so further experiments were made to verify the detector’s stability in the presence of O_2_, N_2_, and CO_2_ gases and the resilience to humidity to simulate the environment of a patient’s breath. The research did not find any serious signal changes due to these non-competent gases, proving the possible application of this system as a diagnostic tool for medical conditions.

- *Other transduction methods*

One particular sensor was based on a CuO-Single-Walled Carbon Nanotubes system, which proved quite sensitive to H_2_S vapors [14]. A remarkably low detection limit of 100 ppb was reported; moreover, the device was easily integrated on an Radio -Frequency Identification (RFID) wearable platform.

### Nanomaterial Production Techniques

Nanomaterial structure deeply influences their macroscopic and microscopic properties. Thus, it is interesting to understand how the process of nanomaterial creation produces a broad range of structures starting from the same original material.

Nanomaterials are a broad class of materials, whose dimensions are typically in the nanoscale range and, in the case of gas sensing, they are utilized for their high surface area. Some examples of nanomaterials include nanowires, nanorods, nanoplates, nanocrystals, and nanoparticles, and they can be obtained through a number of synthesis methods and are generally categorized by two approaches: top–down and bottom–up.

The top–down approach involves the subsequent miniaturization of some bulk materials through a series of steps for the creation of much smaller nanomaterial products. These methods are generally able to control the growth process and define the nanomaterial dimensions very accurately, but they do require specialized equipment and are generally regarded as high-cost methods difficult to scale up. One of the most commonly applied methods is lithography, where well-defined nanomaterials can be obtained employing film exposure and etching. One example of this technique is by using high-resolution electron-beam lithography [15,16,17]. Another common method for this approach is electrospinning [18,19,20]: an electrohydrodynamic method where a charged liquid solution is ejected through a nozzle to the direction of the counter electrode; through evaporation or melt solidification, nanothread-type objects can be created in random orientation.

The bottom–up approach can provide the synthesis of nanomaterials from smaller building blocks, such as atoms and molecules, through various self-assembly processes. These synthesis methods are generally large scale but with less control over the resulting product dimensions. One of the most popular bottom–up synthesis methods of nanomaterials is the hydrothermal method [10,14,21,22]. This synthesis method involves a chemical reaction between different chemical precursors that are dissolved in a liquid solution under vigorous stirring, leading to nucleation and nanostructure formation. Then, the resulting products are processed depending on the needs of the situation and the possibilities of the particular reaction, but it usually involves nanomaterial drying, filtration, and purification from undesired chemical reactants. One of the reasons this technique is so popular is because the resulting nanostructure morphology and properties can often be controlled by varying the synthesis parameters such as used chemical precursors and additives [10,23,24], chemical precursor ratios [25,26,27], growth time [23,26,27,28], and temperature [25,26,29], making this technique quite versatile and universal. Another techniques for the bottom–up approach is the chemical vapor deposition (CVD) [30,31,32,33] technique: synthesis occurs in a high-temperature environment when carrier gases transfer the growth material vapor to the growth surface. The growth surfaces are usually covered in some form of catalyst such as gold, that absorbs the transfer vapor and initiates the growth of nanomaterials, although non-catalytic CVD synthesis is also quite common [34]. One further example is the synthesis of nanomaterials through the use of special templates such as anodic aluminum oxide membranes with well-defined thickness and length of the pores. The resulting nanomaterial product is defined by the dimension of these pores: it is fabricated by filling the pores with some precursor solution, which is then solidified. Then, the template is removed, usually involving a chemical process, to separate the nanomaterials [35,36,37].

Graphite oxide (GO) is a very common material for gas-sensing applications and is synthesized employing the Hummers method [38], which is used with minor modifications for over fifty years. This method is a chemical-based synthesis method that involves the reaction between graphite flakes, NaNO_3_, H_2_SO_4_, and potassium permanganate.

Sacrificial anode electrolysis is another interesting synthesis method for the production of metal and metal oxide colloidal nanoparticles. It involves the electrochemical formation of nanoparticles from a metal source (the sacrificial electrode) in an electrolyte solution. The size, structure, and composition of the resulting nanoparticles can be controlled by varying the current densities of the electrolysis process, the chemical composition of electrolyte solution, and/or by adding chemical stabilizers and surfactants to the reaction media. Furthermore, while performing the process in an inert atmosphere will result in metal nanoparticles, performing it in an oxygen-rich environment will result in metal oxide nanoparticles [39,40,41]. A short recap of the described techniques is presented in Table 1.

## 3. Gas Sensors Based on Polymers

There is a considerable approach for the enhancement of the mechanical strength and characteristics of sensors by combining the organic materials with inorganic counterparts to form composites. Conducting polymers such as polyaniline and polypyrrole have been widely investigated as useful materials for chemical sensors, as they are able to increase the selectivity toward chemical classes of compounds [49]. Conducting polymers are a new class of sensing materials, which can be prepared by a simple chemical oxidative polymerization method. They exhibit reversible pH-induced spectroscopic and gas-induced conductivity changes. In particular, following an interaction with the target gas, the structure of some functional groups of the polymer change, resulting in an overall significant conductivity change of the bulk polymer. Among the conducting polymers, polyaniline (PANI) is frequently used due to its ease of synthesis, environmental stability, and intrinsic redox reaction. To obtain the materials with synergistic or complementary behavior, various composites of polyaniline with inorganic nanoparticles have been synthesized in recent years for the development of novel gas sensors [50,51,52].

In the following, the sensors are classified based on the transduction mechanism.

- *Impedance-based transduction*

It has been demonstrated that polyaniline combined with graphene oxide (GO) and ZnO nanoparticles in a PANI/GO/PANI/ZnO (PGPZ) tetralayer combination showed good gas sensing abilities of ammonia (NH_3_) gas, by measuring changes of impedance in the electrical system [21]. The response of the sensor was evaluated using the formula:

$$Response\;=\;\frac{Z_{g}^{’}}{Z_{air}^{’}}\times 100$$

where *Z*’ is the frequency-dependent real part of impedance in gas and air.

The research was focused on studying different tetralayer combinations for gas sensing and points out that three-level tetralayers, marked as PGPZ3T, when compared to two levels (PGPZ2T) and four levels (PGPZ4T), were the least affected by continuous exposure to NH_3_ and had a stable detection range of 25–500 ppm with a response time of 10–30 s at room temperature.

- *Conductivity-based transduction*

In this class of sensors, the interaction of the sensing element with the target gas causes an overall variation of the resistance of the material. The formula used to calculate the sensitivity of the sensor toward the gas to whom it is exposed is

$$Sensitivity\;\Delta R\left(\%\right)=\frac{R_{g}-R_{a}}{R_{a}}\times \;100$$

where *R_a_* is the resistance measured for the sensor when exposed to air, and *R_g_* is the resistance of the sensor when exposed to target gas.

PANI, PANI/GO, and PANI/GO/ZnO hybrid sensors have been also described, especially in response to a gas mixture, e.g., liquefied petroleum gas (LPG), NH_3_, CO_2_, and H_2_S gases at fixed concentrations, showing that undoped PANI-based sensors have poor selectivity for these gases [22]. However, much better results were achieved by PANI/GO and PANI/GO/ZnO hybrid sensors, with response values being around 2.5–4 times larger for NH_3_ in PANI/GO sensors when compared to other gases and 2–3 times larger in PANI/GO/ZnO hybrid sensors. It is important to point out that PANI/GO sensors were the most sensitive to NH_3_ gas. In addition, PANI sensors did not show full recovery, while PANI/GO and PANI/GO/ZnO provided recovery times of 87, 132, and 78 s, respectively. The response times for all these sensors were 2–5 s with a detection limit of 50 ppm.

**Figure 2:**
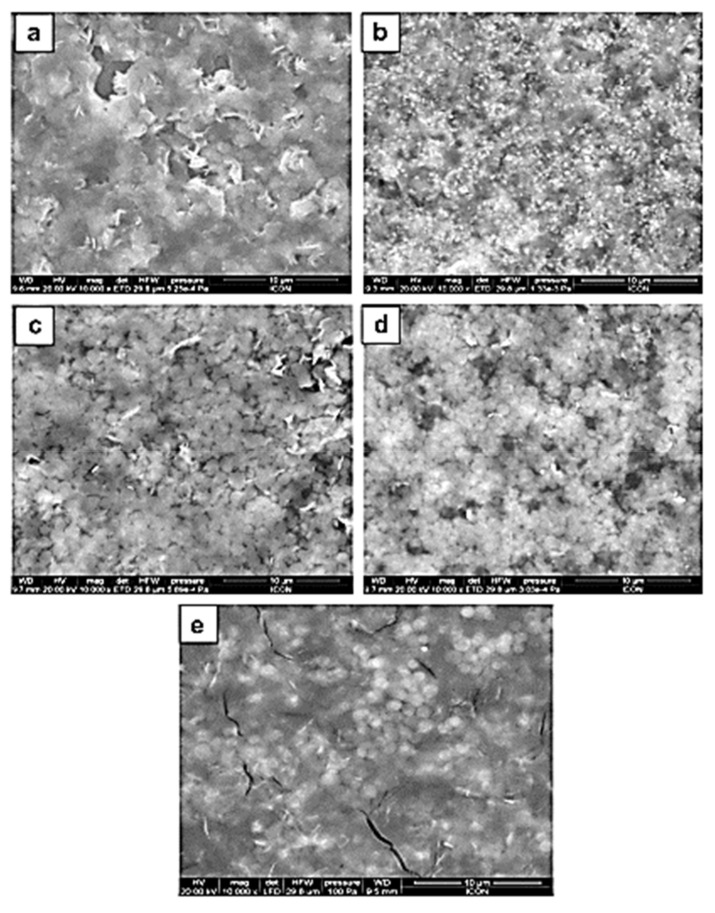
SEM images reported in the work of Patil et al.: (**a**) polyaniline (PANI)–ZnO with 10% of camphor sulfonic acid (CSA); (**b**) PANI–ZnO with 20% CSA; (**c**) PANI–ZnO with 30% CSA; (**d**) PANI–ZnO with 40% CSA and (**e**) PANI–ZnO with 50% CSA. Reproduced from [53] with permission from Elsevier (License number 4930190716578).

A working NH_3_ gas sensor-based PANI/ZnO hybrid film doped with camphor sulfonic acid (CSA) showed good selectivity toward NH_3_ when compared to NO_2_, H_2_S, C_2_H_5_OH, and CH_3_OH gases at a fixed concentration of 100 ppm, with response values being around 13 times larger for NH_3_ gas [53]. PANI/ZnO hybrid films with 50% CSA dopant showed increasing responsivity at room temperature from 10 to 100 ppm for NH_3_ gas, but the sensitivity started dropping after increasing the gas concentration any further. The responsivity of these gas sensors varied from 66–22 s and recovery time was 256–418 s, depending on the gas concentrations. Figure 2 shows the surface images of the CSA-doped PANI–ZnO nanocomposite films. With the increase of CSA dopant, the films became more homogenous, evenly spread out, and, due to the properties of CSA as dopant, more conductive and better responsive to the NH_3_ target gas. Long-term stability tests point out that after 5 days, the sensor does experience responsivity loss from 28.11 to 25.54%, but after that for a total period of 40 days, it did not show any further degradation, indicating the long-term stability of these sensors.

A gas sensor for the detection of NH_3_, HCl gas, and ethanol vapor was also fabricated with the use of polyaniline nanowires grown by a scalable and cost-effective template, with free electrochemical process at site-specific electrode junctions in which the grown nanowire networks connected 2 µm gaps between pairs of Pt electrodes created by lithographic methods [54]. The reported nanowires were uniform in diameter (40–80 nm) and similar in resistance (300–1000 Ω). Before the measurements, the system was doped in 1.0 M HCl solution for the detection of NH_3_ gas and subsequently undoped by 1.0 M NH4OH solution for the detection of HCL gas and ethanol vapor. The system showed an increase in resistance of 1.2 orders of magnitude in 80 s when exposed to NH_3_ gas (100 ppm) and it change by four orders of magnitude in 5 s when exposed to HCL gas (100 ppm) with the lowest concentration of NH_3_ gas 0.5 ppm. Organic vapors such as ethanol were also found to be detectable, and the reproducibility of NH_3_ gas and ethanol vapor detection for this system was proven by repeated exposure to these compounds, but the change in resistivity decreased over time, which was possibly due to the loss of gas and vapor in the open measuring system employed by this study. The selectivity for other types of gases was not carried out in this particular study, but interestingly enough, this sensor showed the ability to detect pH levels of aqueous solutions as well.

Chloroform gas sensors have also been achieved with the use of PANI/Cu hybrid nanocomposite thin films at various concentrations [55] ranging from 10 to 100 ppm by detecting changes in the resistivity of the sample. Chloroform vapor was mixed with hexane gas, and the study does prove that these sensors are capable of distinguishing between pure hexane and hexane/chloroform mix.

- *Optical-based transduction*

Optical properties can also be utilized for the detection of gases [56]. Here, photoluminescence intensity was measured before and after exposing PANI/ZnO nanocomposites to acetic acid, which decreased linearly and could be calibrated for the changes of acetic gas concentration, for concentrations ranging from 1 to 13 ppm at room temperature with two different light sources (520 and 380 nm) using the following equation:

$$Sensitivity=\frac{\left(L_{0}-Lg\right)}{L_{0}}$$

where *L*_0_ and *L_g_* is photoluminescence intensity before and after exposure to the gas, respectively.

The detection limit was found to be 2 ppm and 1.2 ppm for 520 and 380 nm wavelengths respectively, and response of the system was found to be 30 s. The recovery depends on the concentration of the acetic acid, and it was found to be 215–360 s. The stability of this sensor was verified in a 40-day period where the sensor was exposed to 5 ppm of gas, showing that the sensitivity of the system was not seriously affected for 30 days. Sensor selectivity was also tested when water and 100 ppm ethanol was introduced to the gas chamber, but no changes in the signal were detected.

- *Electrochemical-based transduction*

Another research group [57] fabricated a single-use sensor for the detection of NO at very low concentrations for the application of NO as a biomarker for a variety of medical conditions. The sensor active element for detection was a NO-selective membrane–electrolyte film composed of nickel-based porphyrin and Nafion that was applied on top of the Pt electrode and subsequently dried before the detection of NO. This sensor could be used to detect NO not only in the gas phase but also in the liquid phase, and the measurements were carried out with the differential pulse voltammetry (DPV) technique resulting in a calibration curve with equation:

$$y=2.5\times 10^{-9}x+1.4\times 10^{-7}$$

where *x* was the concentration (in ppb) of NO to whom the sensor was exposed, and *y* was the current intensity response measured (expressed in Ampere).

This study was carried out for NO gas phase at the range of 5–25 ppb, but the paper points out that it is not limited to these concentrations; however, the recovery and responsivity for detecting gas-phase NO were not mentioned, and resistivity to other gases such as CO_2_, N_2_, and O_2_ and humidity that would be important in the case of a breath analyzing tool for the diagnosis of various medical conditions was also not mentioned.

### Polymer Synthesis Techniques

The integration of polymer or their composite materials in gas sensing research has been a common practice for quite some time [58]. The most popular for this purpose are conductive polymers due to their metallic or semiconducting electrical properties, simple fabrication process, and low material weight combined with sensitivity to a wide range of gases [21,22,53,54,55,58]. All these properties have made conductive polymers very attractive in the gas sensor industry with application ranging from biosensors [59,60,61] to optical sensors [62,63]. There is a wide range of conductive polymer materials suitable for gas sensing; their main synthetization techniques are electrochemical and chemical polymerization.

The most commonly used method for the fabrication of conducting polymers is electrochemical polymerization [64]. This method involves oxidation or reduction of the monomers, inducing polymerization on one of the electrochemical cell electrodes, thus inducing permanent changes in the electrical conductivity, optical activity, corrosion stability, and other properties of the resulting polymer product [65]. The conductivity of the polymer produced with this method depends on numerous parameters, such as the used aqueous or organic electrolytic solution, the oxidation or reduction rates, voltages, currents, and materials used for the reaction electrodes. This in turn gives the ability to “tune” the properties of the polymer as desired. Conductive polymer such as polyaniline [66], polypyrrole [67], polypyridyl [68], and polyindole [69] are commonly fabricated using this technique.

Chemical polymerization, on the other hand, does not use electrical currents and voltages during the polymerization process, instead inducing changes to the polymer chains with the help of chemical reagents. For example, polyaniline can also be synthesized by adding ammonium peroxydisulfate oxidant to a 1 M HCL solution of aniline monomers to initiate polymerization [70]; polypyrrole can be obtained by adding iron (III) chloride to a solution of nitromethane and pyrrole monomers [71]; polythiophene can be obtained by polymerizing 3,4-ethylenedioxythiophene with sodium alkylnaphthalene-sulfonate and iron (III) sulfate [72].

In chemical polymerization temperature, proportions of chemical reagents and the presence of other additives such as organic dopants, surfactants, and polyelectrolytes all have an impact on the resulting polymer structures, allowing for some degree of control of the product properties [70]. A brief recap of the cited methods for conductive polymer synthesis are presented in Table 2.

## 4. Gas Sensors Based on Carbon Nanomaterials

Carbon nanostructures have always had an important role in the development of sensors, thanks to their unique chemical, electrical, and mechanical properties. However, for the most part, their role has been restricted as a support material for the sensing element. In gas sensing, there are some examples of carbon nanostructures used as a direct sensing element for various gasses. The sensors are classified based on the transduction methods, as follows.

- *Conductivity-based transduction*

In this class of sensors, the interaction of the sensing element with the target gas causes an overall variation of the resistance of the material. The formula used to calculate the sensitivity of the sensor toward the gas to which it is exposed is

$$Sensitivity\;\Delta R\left(\%\right)=\frac{R_{g}-R_{a}}{R_{a}}\times \;100$$

where *R_a_* is the resistance measured for the sensor when exposed to air, and *R_g_* is resistance of the sensor when exposed to target gas.

A gas sensor for the detection CO_2_ was developed employing a multiwall carbon nanotube/Al_2_O_3_ composite [73]. The aim of the research was to assemble a sensor capable of monitoring CO_2_ concentrations variations in air, since the impact of CO_2_ on the global warming is one of the biggest concerns today. It was found that exposure to CO_2_ gas at various concentration levels at room temperature has an increase in the resistance of the sensing element made of multiwalled carbon nanotubes (MWCNTs) immobilized in an alumina sol. In particular, a MWCNTs concentration of 1.5%_wt_ was the most optimal for sensing with a response of 7.3% at a concentration of 450 ppm CO_2_ gas with linear response in the range of 50–450 ppm. However, the sensor was found to have recovery and stability problems with a recovery time of around 25 min and drift in the baseline resistance due to the insufficient desorption of CO_2_ gas on the sensing element. The recovery with thermal energy stimulation of the sensing element was successful with a complete recovery in 14.15 s.

Another carbon material-based gas sensor was fabricated employing as a sensing element hydroxyl-functionalized graphene quantum dots (average size 5 nm) deposited on nickel electrodes [74]. This sensor was able to detect NH_3_ in a gas concentration range of 10–500 ppm. The sensor also showed good response and recovery times of 64 and 69 s at 500 ppm, respectively. The selectivity was also tested toward possible interference such as O_2_, formalin, ethanol, methanol, toluene, acetone, dimethylformamide (DMF) gases, and vapors at a fixed concentration of 500 ppm. The only interference was found from DMF and O_2_.

In another work [75], a room temperature H_2_ gas sensor based on acid-treated carbon nanotube (CNT) nanoarrays was assembled. The electrical response of the sensor, although at a quite large gas concentration (from 20,000 to 200,000 ppm) was found to be 2–8%. The sensor also showed good selectivity against acetylene, methane, CO, and CO_2_. The authors point out that H_2_SO_4_ acid solution treatment of the CNT arrays for 1 h improved the gas sensors conductivity by more closely interconnecting the CNT fibers, thus creating better electrical pathways and also creating oxygen defects on the surface that allowed for the exclusive detection of only H_2_.

A single layer graphene/Au electrode was proposed as an NH_3_ conductivity gas sensor [76]. The response values peaked at around 4.5, 6.0, 7.5, and 9.5% after exposure time of 8 min at gas concentrations of 100, 200, 400, and 800 ppm, respectively. The stability of the sensor was also tested with baseline resistance not changing more than 1.5% after repeated measures, indicating good long-term reliability.

- *Optical-based transduction*

An optical-based graphene quantum dot (GQD) gas sensor for the detection of CO_2_ was proposed with GQD of sizes around 10–20 nm [77]. The sensing was based on the changes in the light absorbance after exposure to target gas, and the authors performed sensor response tests in the range of 100–1000 ppm. The response relationship to gas concentration could be expressed by the following exponential equation:

$$A=A_{0}\exp[{\left(\alpha N\right)}^{\beta }]$$

where *A*_0_ is initial optical absorption, *A* is optical absorption at the concentration of N, and α and β are constants.

In Table 3 a comparison between the metal oxide-based, polymer-based and carbon-based sensor is presented. Here listed are the main advantages and disadvantages of each class of sensors, together with possible solutions to improve them.

## 5. Biological Elements for Gas Sensing

In recent years, sensors employing sensing elements derived from living organisms have gained increased attention from the research community, also in the field of biosensors for gas detection and quantifications. The biosensors are classified based on the biological element as follows.

- *Biosensors based on enzymes*

The main examples are gas sensors based on enzyme and/or enzyme cycles. As an example, a biosensor for ethanol vapors was realized exploiting chromatography paper on top of an electrode to immobilize the enzymes alcohol oxidase and horseradish peroxidase [78]. In detail, in the presence of oxygen, alcohol oxidase oxidizes alcohol to acetaldehyde and hydrogen peroxide. Then, hydrogen peroxide is used by horseradish peroxidase to reduce Fe(CN_6_)^4−^ to Fe(CN_6_)^3−^; Fe(CN_6_)^3−^ is again oxidized back to Fe(CN_6_)^4−^ during the amperometric measurement, thus closing the cycle. The system had a limit of detection of ethanol vapor of 50 ppm within a linear range of up to 500 ppm.

Another biosensing solution is the so-called “bio-sniffers”. These systems are based on a fiber-optic probe at the end of which the enzymatic reaction takes place, resulting in a light spectral change that can be measured corresponding to the target analyte concentrations. As an example, a membrane containing the formaldehyde dehydrogenase enzyme was attached at the end of the probe, with a microcapillary system for water and nicotinamide adenine dinucleotide (NAD^+^) circulation [79]. When formaldehyde enters the system, it is oxidized in the presence of NAD^+^ by the enzyme formaldehyde dehydrogenase to formate, while NAD^+^ is reduced to NADH. The optical fiber is used to excite the system with UV light and monitor back the fluorescence produced by the NADH produced (emission peak at 335 nm). The system showed a limit of detection of 2.5 ppb within a linear range up to 10 ppm. It was tested against various common volatiles such as acetaldehyde, acetone, benzene, methanol, and ethanol, but none of these recorded a signal above baseline noise, demonstrating that the sensor is highly selective.

A sensor based on the “bio-sniffer” concept was also developed to monitor isopropanol in breath as a marker for some chronic diseases (such as liver disease, chronic pulmonary obstruction, and lung cancer) [80]. Here again, an optical fiber was employed; this time, secondary alcohol dehydrogenase enzyme was immobilized at the end of the optical fiber, with a microcirculation system for NAD^+^. In this case, at the sensing end of the probe, isopropanol was transformed to acetone by secondary alcohol dehydrogenase in the presence of the coenzyme NAD^+^, which in turn is reduced to NADH. The optical fiber was used to guide the excitation UV light and guide back to the detector the fluorescence of the NADH produced in the reaction. The sensor was able to provide a limit of detection of 1 ppb within a linear range up to 9060 ppb. The biosensor was tested against common interferences; significant interferences were found when the sensor was exposed to molecules similar to the target, such as 1-propanol and 1-butanol.

Butyrylcholinesterase was also exploited for the design of an electrochemical sensor for nerve agents detection such as VX and Sarin, based on screen-printed electrodes (SPEs) modified with Prussian blue as an electrochemical mediator [81]. Usually, nerve agents are liquid at room temperature but, being extremely toxic for human health even at extremely low concentrations, the authors of the papers assembled a sensor for their detection in the gas phase. In this study, paraoxon was used as simulant and detected as low as 5 ppb (within a linear range up to 100 ppb), Sarin was detected at concentrations of 12 ppb (within a linear range up to 20 ppb), and VX was detected at concentrations of at 14 ppb (within a linear range up to 150 ppb). These results show that the developed SPE-based platform could be a useful tool for the detection and identification of such gases by first responders and military personnel.

Another solution for the monitoring of cholinesterase inhibitors is proposed in another study [82] in which butyrylcholinesterase is paired with horseradish peroxidase and encapsulated in carbon nanotubes, which are adhered to a SPE. This solution allowed having all the enzymes and mediators relegated in close proximity without the need for other immobilization techniques, which could inhibit the activity of one of the two enzymes. With this system, the authors detected malathion (a cholinesterase inhibitor) at concentrations as low as 6 ppb, within a linear range up to 25 ppb. Furthermore, they proved that their sensor could be freeze stored for up to six weeks and used multiple times with minimal performance degradation. As seen before, formaldehyde is often the target gas for detection, since its presence in work environments, even at low concentrations, can cause long-lasting damage to workers. A two-enzyme cycle system has been proposed for its detection [83]. The first enzyme is formaldehyde dehydrogenase which, in the presence of coenzyme NAD^+^, produces formic acid and coenzyme NADH. The reduced coenzyme NADH is used by the enzyme diaphorase in conjunction with its substrate (tetrazolium salt WST-8) to produce yellow WST-8 formazan and NAD^+^. Thus, the coenzyme NAD^+^ is once again available for the first cycle, while the concentration of formaldehyde can be calculated by measuring the change in the absorbance of WST-8 formazan at 460 nm. The resulting sensor could detect concentrations of formaldehyde as low as 1.5 ppb within a linear range up to 80 ppb. One of the degradation products of formaldehyde is formic acid, whose exposition over long periods can produce long-lasting damages to the human organism. A sensor based on an SPE modified with formate dehydrogenase and coenzyme NAD^+^ was assembled to selectively quantify formic acid down to 16 ppb, with only small interferences from acetic acid [84]. The sensor was also employed in a real environment (a factory in which formic acid-based glues were used), showing the capability to quantify the amount of formic acid in the air without suffering from interferents.

- *Biosensors based on non-enzymatic proteins and structures*

Although enzymes are one of the most common occurrences in gas biosensors, they are not the only option available as recognizing elements. In a paper [85], single DNA strands immobilized on single-walled carbon nanotubes were used to selectively quantify some compounds of interest. In particular, it was intended to monitor ethylhexanol, linalool, tetradecene, and phenylacetaldehyde, since these compounds serve as secondary biomarkers for the detection of citrus trees infected by Huanglongbing disease (also known as citrus greening or yellow shoot) during the asymptomatic stage. This disease is fatal for the affected trees if not cured early. This sensor was able to detect the compounds of interest over a wide range of concentrations, while at the same time discriminating the signals coming from other chemicals, thanks to a principal component analysis technique followed by a neural net fitting. Furthermore, the combination of inorganic nanomaterial and organic polymers can give rise to interesting combinations: such as the case of a reported biosensor based on chitosan immobilized on reduced graphene oxide [86]. The device proved to be useful to quantify acetone in human breath, which is one key early warning marker of diabetes, at concentrations as low as 10 ppm. Another molecule of interest employed is cytochrome c. This molecule can be easily used in the redox process since it contains an Fe atom that easily interchanges between its 2^+^ and 3^+^ states. Thus, cytochrome c was used [87] to demonstrate how a gas–liquid sensor could be assembled: cytochrome c, bound to SnO_2_ nanosphere, which in turn is deposited on fluorine doped tin oxide (FTO), is first oxidized, bringing the Fe atom to its 3^+^ state. In correspondence to this, a light absorbance at 550 nm was observed, which is characteristic of cytochrome c. When the target gas was fluxed, in this case, methanethiol, an increase in absorbance was observed, corresponding to a reduction of cytochrome c Fe to its 2^+^ state and corresponding methanethiol oxidation. By further electrochemically oxidizing the sensor after exposure to the target gas, it was possible to return cytochrome c to its oxidized state, thus regenerating the sensor. One interesting application of bioelements to develop biosensors comes from the use of complex biological structures naturally present in the cell bilayer of certain organisms. In a work [88], the sensing element employed is the olfactory receptors of mosquitoes, which are embedded in a bilayer lipid membrane. This membrane was used to separate two solutions: one poor in ions, the other rich. The receptors were specific to 1-octen-3-ol, and when in the presence of the target gas, the variation in the conformation of the receptor caused passages in the lipid bilayer membrane to open, allowing ions to flow between the two solutions. The ion flow between the two solutions was monitored by measuring the electrical current flow between the two solutions. With this system, which proved quite selective against similar molecules such as octanol and octanone, this biosensor was able to detect concentrations of 1-octen-3-ol as low as 10 ppb.

- *Biosensors based on live cells*

**Figure 3:**
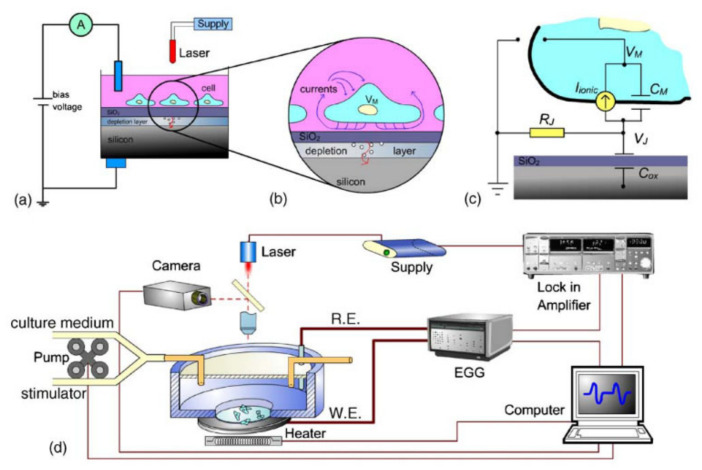
An example of a sensor-based on olfactory cells (**a**) with a detail of cell interfacing with transducer (**b**) and circuitry involved (**c**). A broader scheme of the experiments is reported (**d**). Reproduced from [91] with permission from Elsevier (License number 4930191079175).

The creation of a gas biosensor that uses living cells has always proved to be more difficult than other bio-approaches. Since the sensing element is a living being, it needs to be fed and cared for (for example, providing micronutrients and removing wastes produced) to not introduce unknown variables in the measured signal. Nonetheless, a biosensor based on a recombinant bioluminescent *Escherichia coli* harboring a lac::luxCDABE fusion is reported [89]. In this biosensor, the bacteria whole cells were immobilized on the sensor surface through lysogeny broth (LB) agar. When the sensor was exposed to vapors toxic to the *E. coli*, a reduction in the luminescence signal was recorded. Using benzene as the target gas to detect, the luminescence reduction was proportional to vapor concentration, and the sensor was able to detect benzene concentrations as low as 48 ppm. Another approach on this line regards the use of *Pseudomonas putida* cells modified to carry NAH7 plasmid and a gene fusion between the sal promoter and the luxAB genes [90]. The so-modified cells were used to detect naphthalene vapor arising from liquid samples. The biosensor assembled proved to be sensitive to the target gas, with a luminescence response proportional to the naphthalene concentration and a limit of detection of 64 ppb. On the other hand, the sensor showed small but significant responses also from other chemicals such as DMSO and methylated analogs of naphthalene. Finally, an interesting solution in the development of a biosensor using living cells regards the growth of olfactory receptor neurons and olfactory bulb cells on a semiconductor chip to simulate the human olfactory system and to detect the potential changes in the olfactory neurons in response to volatiles exposition [91]. As shown in Figure 3, when the olfactory cell interacts with the target analyte, it produces variations in the local concentrations of K^+^ and Na^+^ ions (Figure 3a,b). These variations are measured by a semiconductor layer at a fixed electrical potential acting as a transducer (Figure 3c). In particular, the biosensor was able to detect acetic acid vapor at concentrations ranging from 1 to about 60 ppm.

### Bio-Element Immobilization

One of the first points to consider when evaluating bio-based gas sensors is the immobilization technique employed to integrate the sensing element into the sensor surfaces. While non-bio-based sensors can use high temperatures and other extreme conditions to permanently bind the substances, bioelements require more care and thus different approaches. It should be kept in mind that not all techniques are optimal for the immobilization of all bioelements. One common technique is to form covalent bonds between the bioactive element and the sensor transducer. With this technique, the bioelement is covalently bound to the surface of the sensor. The binding happens through specific couples of functional groups present both on the bioelement and the sensor surface. The main advantage is that since covalent bonds are quite strong, it is difficult for the bioelement to leave the sensor surface during storage and/or use. This technique has been successfully employed to bind laccase on citric acid-functionalized biochar [92] and in the creation of resistant but permeable hydrogel to entrap enzymes. As an example, pepsin was immobilized on poly-(ethylene glycol) dimethacrylate via UV-initiated radical polymerization of a mixture of enzyme and monomer [93].

The main drawback of the covalent immobilization is that the bioelement needs to have an exposed functional group that is useful to perform the covalent binding and that the resulting binding should not interfere with the bioelement performance. Another suitable technique is to entrap the bioelement in a matrix. This technique, widely used in the production of biosensors in general, is finding applications also in the production of biosensors for gas sensing. With this technique, the main limitation is that the entrapment matrix should be tight enough to hold the bioelement but at the same time wide enough to permit gas exchange with the environment. These limitations have been solved by entrapping the bioelement on natural high-porous substrates such as mesoporous carbon nanospheres [94] or in aerogels [95]. One such example is an aerogel [96] made of a mixture of platinum and carbon to assemble an acetylcholinesterase biosensor for the detection and quantification of organophosphorus pesticides. One innovative technique for bioelement deposition is the electrospray ionization (ESI) technique, which borrows the ionization procedure of mass spectrometry to create charged droplets of biomaterial that, upon impact with the sensor surface, create strong bonds [97,98]. Although the technique has been tested only using enzymes (laccase oxidase in particular), it seems promising for use with larger objects (such as DNA strands or even live cells).

In Table 4, some examples of bio-element immobilization are reported with some possible applications.

## 6. Analytical Sensing Properties Comparison of Reported Gas Sensors

In Table 5, the reported gas sensors have been compared in terms of technology employed for sensing, target gas(es) to which they are tuned for, limit of detection and upper limit of linearity for their target gas, as well as their selectivity in terms of response toward possible interferents. From the data reported in the table, it appears that sensors based on inorganic materials have been developed to detect a wide array of substances, typically small molecules that are toxic to human health. They have great stability over time, but often, they suffer from interference from molecules that are chemically similar to the target ones. Limits of detection are usually around the part per million (ppm), with the upper limit of linearity being two or three orders of magnitude bigger. On the other hand, gas biosensors are much more specific for their target molecules and suffer much less from the interference of chemically similar substances. The detection limits are in the range of part per billion (ppb), but the ranges of linearity are usually smaller compared to the inorganic ones.

## 7. Conclusions

Research in gas sensors and biosensors has well progressed in recent years. Most gas sensor detection mechanisms are based on electrical changes when exposed to increasing concentrations of target gas, but sometimes, optical changes and biological/chemical processes can also be utilized for the same purpose. Both of these systems show their intrinsic advantages/disadvantages. Regarding inorganic-based gas sensors, notable improvements have been achieved regarding lowering the detection limit of the developed platforms, but there is still room for improvement in other areas such as limiting the sensor response only to target gas, improving the stability of sensors after continuous exposure to the target, and increasing the long-term stability.

Although most efforts have been centered on the development of gas sensors based on non-organic materials, biological gas sensors have also shown great results and sometimes even better detection limits when compared to the latest gas sensors based on inorganic nanomaterials, proving that biological gas sensors are highly competitive and can have lots of prospective applications in the future. Furthermore, they often show high selectivity toward target analytes.

If sensors based on non-biological materials have simpler fabrication stages and are often easier to mass produce, biological material-based sensors have much more complicated fabrication procedures, such as immobilization of the biological material. Furthermore, they have low availability for mass production and are limited to certain environmental conditions to prevent the degradation of biological material employed. Moreover, not all biological materials commonly used for the development of gas sensors fit well for sensing analytes in the gas phase. A comparison between the two types of gas sensors is presented in Table 6.

**Figure 4:**
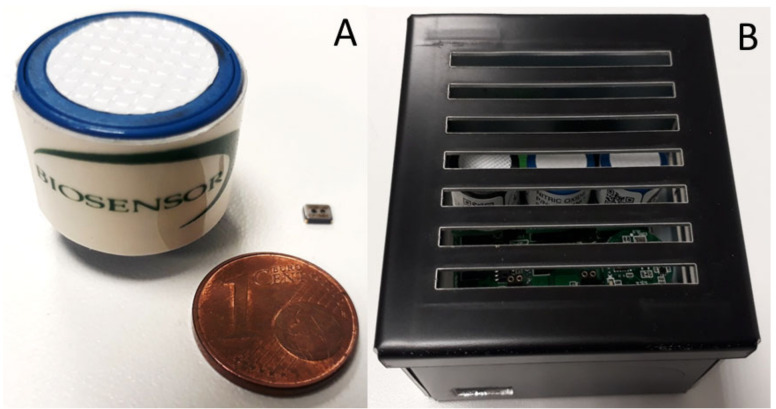
(**A**) Electrochemical and metal oxide semiconductor sensor used by Biosensor Srl to develop (**B**) multigas sensor array to detect and quantify NOx and acetic acid.

Regarding their application in real fields, gas sensors, both inorganic and bio-based, are gaining traction as key instruments in many areas. One particular example is in cultural heritage protection as exemplified by the European project Nemosine [99]: the Biosensor Srl company has developed an array that integrates sensors based on nanomaterials for NO, NO_2_, (electrochemical), and for acetic acid (resistive, based on coupled zinc oxide tin oxide/zinc oxide nanostructured sensors) (Figure 4A). The array was tested to monitor its performance when challenged with cross interferences of secondary volatile co-pollutants produced by the degradation process of films but not involved in further degradation processes (such as medium-chain alcohols, benzoquinones, phenols). Then, the arrays, which have a small total volume dimension (Figure 4B) were used to analyze, under controlled laboratory conditions, cellulose acetate and nitrocellulose real films.

Further developments are expected on both types of gas sensors, but also the birth of a third class of hybrid sensing solutions, in which nanostructures are used in the role of both scaffold and transducer for biological element [100,101], offering long-lasting sensing, in a wide range of applications with optimal sensibility and selectivity performances.

## Figures and Tables

**Table 1:** Nanostructure synthesis methods.

Approaches	Fabrication Method	Nanostructure Example	Reference
**Top–Down**	High-resolution lithography	Nanowires	[17]
		Nanoparticles	[42]
		Nanorods	[43]
		Nanodisks	[44]
		Nanoarrays	[45]
	Electrospinning	Nanofiber	[18]
		Nanowires	[20]
**Bottom–Up**	Chemical vapor deposition	Nanowires	[31,32]
		Nanoribbons	
		Nanorods	
		Nanoparticles	
	Anodic aluminum oxide membranes	Nanowires	[46]
		Nanoarrays	
		Nanopillars	[47]
		Nanotubes	[48]
	Hydrothermal synthesis	Nanoparticles	[21]
		Nanotubes	[14]
		Nanoflakes	[10]
	Hummers method	Graphene oxide nanostructures	[38]
	Sacrificial anode electrolysis	Metal nanoparticles	[39,40,41]
		Metal oxide nanoparticles	

**Table 2:** Conductive polymer synthesis techniques.

Polymerization Method	Polymerization Products	Reference
**Electrical Polymerization**	Polyaniline	[22,66]
	Polypyrrole	[67]
	Polypyridyl	[68]
	Polyindole	[69]
**Chemical Polymerization**	Polyaniline	[22]
	Polypyrrole	[70]
	Polythiophene	[72]

**Table 3:** Advantages and disadvantages of metal oxide, polymer, and carbon nanomaterial-based gas sensors.

Metal Oxide-Based Sensors		Polymer-Based Sensors		Carbon-Based Material Sensors	
Pros	Cons	Pros	Cons	Pros	Cons
High surface-to-volume ratio, large surface area	Require high temperature to work	Operate at room temperature	Response is often unspecific (toward “class” of gasses instead of one)	High surface-to-volume ratio, large surface area	Possible sensing material agglomeration
Can be synthetized in different shapes to tune response to target gas	Response often degrades over time when continuously exposed to gasses	Simple fabrication process	Response may strongly degrade if exposed to temperatures significantly different from RT	Operate at room temperature	Limited detection range
	Possible sensing material agglomeration				May have limited gas absorption and non-linear sensor response
Solutions		Solutions		Solutions	
“Doping” using other metal/metal oxides/polymers to create multi metal or hybrid systems, lowering operating temperature, increasing selectivity and/or lifetime		“Doping” and/or modification using other materials (i.e., carbon structures, metal oxides) to improve selectivity of response		Improved deposition techniques and nanomaterial functionalization (i.e., other carbon-based nanostructures and/or metal oxide nanoparticles) to improve detection range and linearity of response	

**Table 4:** Biomediators immobilization techniques.

Immobilization Method	Immobilization Surface	Reference
**Covalent binding**	Citric acid-functionalized biochar	[92]
	poly-(ethylene glycol) dimethacrylate	[93]
**Matrix entrapment**	Carbon nanospheres	[94]
	Aerogels	[95,96]
**Electrospray ionization**	Glassy carbon electrodes	[97,98]

**Table 5:** Sensing properties of reported gas sensors.

Type of Sensing Element	Sensing Element	Target Gas	Working Temperature of Sensing Element (°C)	Interferents	DATA		Reference
					Limit of Detection (ppm)	Upper Limit of Linearity (ppm)	
**Resistance variation of nanostructured material**	TiO nanocrystal	Benzaldehyde	300	No interference from EtOH, acetone, benzene, toluene, benzyl alcohol, methanol, ammonia gas	10	800	[10]
**Metal oxide thin film**	Single crystalline In_2_O_3_ thin films doped with Mg	Ozone	Nd	Nd	0.05	Nd	[11]
**Thin hybrid film for SPR**	TiO_2_/Au hybrid	Formaldehyde	Room temperature	No interference from CO_2_, H_2_O, and N_2_	0.2	3.5	[13]
**Metal oxide pure and mixtures**	ZnO, SnO ZnO-SnO	1-butanol	350	Nd	ZnO: 0.05 SnO: 0.1 ZnO–SnO: 0.025	Nd	[12]
**RFID platform with metal oxide–nanotube sensor**	SWCNT decorated with Cu nanoparticles	H_2_S	Room temperature	Nd	0.1	50	[14]
**Layer-by-layer structure**	PANI/GO/PANI/ZnO	NH_3_	Room temperature	Nd	25	500	[21]
**Mixed nanocomposites**	Polyaniline/graphene oxide/ zinc oxide	NH_3_	80	No interference from liquid propane gas, CO_2_, H_2_S	50	1000	[22]
**Spin-coated composite material**	Camphor sulfonic acid doped polyaniline–zinc oxide nanocomposites	NH_3_	Room temperature	No interference from NO_2_, H_2_S, ethanol, methanol	10	100	[53]
**Conductive nanowires**	Polyaniline nanowires	NH_3_	Room temperature	HCl, ethanol, polar organic vapors	0.5	Nd	[54]
**Chemically synthesized nanocomposite**	Cu/PANI	Chloroform	Room temperature	No interference from hexane	10	100	[55]
**Nanostructure–nanowire composite**	ZnO/PANI	Acetic acid	Room temperature	No interference from H_2_O, ethanol	1.2	10	[56]
**Ni (II) tetrakis (3-methoxy-4-hydroxyphenyl) porphyrin selective for target gas**	Thin-film platinum-based electrochemical sensor	NO	Room temperature	Nd	0.005	0.025	[57]
**MWCNT sensor for CO_2_ quantification**	MWCNT in alumina sol	CO_2_	Room temperature	Nd	50	450	[73]
**Hydroxyl edge-functionalized graphene quantum dots**	Modified graphene quantum dots on nickel electrodes	NH_3_	Room temperature	No interference from formalin, ethanol, methanol, toluene, acetone; small interference from O_2_, dimethylformamide	10	500	[74]
**Carbon nanotube yarn**	Acid-activated carbon nanotubes	H_2_	Room temperature	No interference from acetylene, methane, CO, and CO_2_	20,000	200,000	[75]
**Single layer graphene/Au electrode**	Single-layer graphene film	NH_3_	Room temperature	Nd	100	800	[76]
**Optical-based graphene quantum dot**	Graphene quantum dots	CO_2_	Room temperature	Nd	100	1000	[77]
**Chromatography paper as enzyme supporting and a liquid phase layer on top of electrode**	Alcohol oxidase-horse radish peroxidase couple	Ethanol	Room temperature	Nd	50	500	[78]
**Fiber-optic biochemical gas sensor (Bio-Sniffer)**	Fluorescence of NADH produced by formaldehyde dehydrogenase	Formaldehyde	Room temperature	No interference from acetaldehyde, acetone, benzene, methanol, ethanol	0.0025	10	[79]
**Fiber-optic biochemical gas sensor (Bio-Sniffer)**	Fluorescence of NADH produced by secondary alcohol dehydrogenase	Isopropanol	Room temperature	1-propanol, 1-butanol	0.001	9.060	[80]
**Amperometric biosensor**	Butyrylcholinesterase inhibition	Nerve agents (Sarin)	Room temperature	Nd	Paraoxon 0.005 Sarin 0.012 VX 0.014	Paraoxon 0.100 Sarin 0.020 VX 0.150	[81]
**Sensing element encapsulated in peptide nanotubes and Nafion**	Butyrylcholinesterase coupled with horseradish peroxidase	Malathion	Room temperature	Nd	0.006	0.025	[82]
**Enzymatic cycling system**	NAD^+^ coupled with WST-8 and diaphorase enzyme	Formaldehyde	Room temperature	Acetaldehyde, methanol, ethanol, acetone, formic acid	0.0015	0.08	[83]
**Electrochemical sensor**	Formate dehydrogenase	Formic acid	Room temperature	No interference from methanol, formaldehyde, small interference of acetic acid	0.016	Nd	[84]
**Modified single-wall carbon nanotubes**	Single-strained DNA	Ethylhexanol, linalool, tetradecene, and phenylacetaldehyde	Room temperature	Interferent VOC discarded through PCA	Nd	Nd	[85]
**Composite film-based sensor**	Chitosan-reduced graphene oxide	Acetone	Room temperature	Small interference from ethylene, formaldehyde, ethanol, methane, and carbon monoxide	10	Nd	[86]
**SnO2-layer on fluorine-doped tin oxide (FTO)-coated glass**	Cytochrome c	Methanethiol	Room temperature	Nd	Nd	Nd	[87]
**Reconstructed bilayer lipid membrane**	Mosquito olfactory receptors	1-octen-3-ol	Room temperature	No interference from octanol, octanone	0.01	0.2	[88]
**Whole-cell biosensor based on recombinant *E. coli***	Lac:luxCDABE fusion	Benzene	Room temperature	Nd	48	Nd	[89]
**Bioluminescent biosensor**	*P. putida* modified pPG7	Naphthalene	Room temperature	Small responses from DMSO, various methylated naphthalenes	0.064	Nd	[90]
**Light-addressable potentiometric sensor**	Olfactory receptor neurons and olfactory bulb cells	Acetic acid	Room temperature	Nd	1.19	59.5	[91]

Nd: Not declared in the article.

**Table 6:** Comparison between gas sensors based on non-biological and biological sensing material.

Non-Biological Sensing Material		Biological Sensing Material	
Pros	Cons	Pros	Cons
Cheap to produce	Often quite non-specific towards target gas	Bio element is often quite specific for target molecule	Sensor must be stored at a fixed condition to prevent bio element degradation
Minimal maintenance of finalized sensor	Require complex post-processing of obtained signal to eliminate noise and/or interferents	Detection can happen at extremely low levels (ppb/ppt)	Bio element may degrade over time, influencing the sensor response
Can be easily integrated into electronic systems	Require material often toxic and/or highly costly	Material to assemble sensors has low environmental impact	Not all bio elements work well for the recognition of gas-phase targets
Can be easily mass-produced	Can be difficult to separate signal from analyte from degradation of sensing material	Genetic editing may render biosensor even more specific and/or sensible for target	Procedures involved in sensor creation may be difficult to replicate in mass production
